# Statistical Evidence for Bimodal Age Distribution in Pediatric Orchiopexy: Support for Congenital and Acquired Cryptorchidism Subtypes

**DOI:** 10.7759/cureus.98672

**Published:** 2025-12-07

**Authors:** Kensuke Ohashi, Shinsuke Yoshizawa, Keita Kogure

**Affiliations:** 1 Department of Urology, Saitama Children's Medical Center, Saitama, JPN; 2 Department of Pediatric Urology, Saitama Children's Medical Center, Saitama, JPN

**Keywords:** bimodal distribution, cryptorchidism, gaussian mixture model, orchiopexy, pediatric urology

## Abstract

Objective: This study aims to characterize the age distribution of orchiopexy procedures and assess whether bimodal patterns exist that may correlate with hypothesized congenital and acquired cryptorchidism subtypes.

Materials and methods: We analyzed age-at-orchiopexy data from 659 consecutive patients (ages zero to 18 years) at a single tertiary pediatric center. Bimodality was assessed using (1) Hartigan's dip test for unimodality, (2) kernel density estimation with bootstrap mode stability analysis, and (3) Gaussian mixture modeling with information criterion-based model selection.

Results: The dip test decisively rejected unimodality (dip = 0.134, p < 0.001). Gaussian mixture modeling strongly favored a two-component model over unimodal (ΔBIC = 407.7). The optimal model identified an early component (mean = 1.31 years, SD = 0.80, weight = 54.2%) and a late component (mean = 6.28 years, SD = 3.35, weight = 45.8%), with an intersection at 3.8 years. Bootstrap analysis confirmed a stable bimodal structure.

Conclusions: Age-at-orchiopexy demonstrates statistically robust bimodality, with early (1.31 years) and late (6.28 years) components. This bimodal pattern is consistent with, and may correlate with, clinically hypothesized congenital and acquired cryptorchidism subtypes, but it does not by itself prove distinct biological entities. The intersection at 3.8 years may offer a quantitative reference point for age-specific management strategies and surveillance protocols and should be interpreted as hypothesis-generating rather than prescriptive.

## Introduction

Cryptorchidism is clinically conceptualized as comprising congenital and acquired subtypes with distinct pathophysiology, natural history, and management implications [1]. Congenital undescended testes result from failed descent during fetal development, typically presenting in infancy and requiring early intervention to optimize fertility and reduce malignancy risk [2]. Acquired cryptorchidism involves secondary ascent of previously descended testes, commonly detected during school-age examinations [3]. This clinical distinction between congenital and acquired presentations is fundamental to pediatric urology practice and guides surgical decision-making [4].

Despite widespread clinical acceptance of this two-subtype paradigm, formal statistical validation of bimodality in age-at-orchiopexy distributions has been limited. Previous studies relied primarily on descriptive histograms or clinical impressions rather than rigorous statistical testing. The absence of quantitative evidence supporting the bimodal hypothesis represents a methodological gap, particularly given the profound implications for disease classification and management protocols.

We aimed to characterize the age distribution of orchiopexy procedures in a large consecutive cohort and to assess whether bimodal patterns exist using complementary analytical approaches such as Hartigan’s dip test for formal rejection of unimodality, kernel density estimation with bootstrap stability assessment, and Gaussian mixture modeling with information-criterion-based model selection. Our hypothesis was that age-at-orchiopexy data from a large consecutive cohort would demonstrate statistically significant bimodality, with peaks in early childhood and school age that are consistent with clinically hypothesized congenital (early) and acquired (late) presentations, rather than definitively proving distinct biological subtypes.

## Materials and methods

This study was conducted at a single tertiary pediatric center (Saitama Children’s Medical Center, Saitama, JPN) over five years from April 2019 to December 2024. It received approval from the Saitama Children’s Medical Center Institutional Review Board (approval no. 2025-02-002).

Inclusion and exclusion criteria

Patients aged zero to 18 years who underwent orchiopexy at Saitama Children’s Medical Center between April 2019 and December 2024 were included in this study. Each patient was counted once at the patient level, regardless of laterality. For bilateral cases, the age at the first orchiopexy within the study period was used as the age-at-orchiopexy for analysis.

Patients with incomplete age data, prior orchiopexy at another institution, or a diagnosis unrelated to cryptorchidism were excluded from analysis. Revision orchiopexy procedures and secondary operations on the same testis were excluded; only primary orchiopexy procedures during the study period were included in the analysis. Cases were considered 'unrelated to cryptorchidism' and excluded if orchiopexy was performed for other indications, such as testicular torsion, neoplasms, or trauma.

Data collection

This study did not adjust for potential confounding factors. All cases of orchiopexy, including those with genetic disorders, were included in the analysis. Similarly, birth weight and gestational age were not adjusted for in the statistical analysis. All consecutive orchiopexy cases meeting the inclusion criteria during the study period were identified retrospectively from the institutional electronic medical record system, ensuring a comprehensive and unbiased sample.

Primary analysis

Bimodality Testing

Hartigan’s dip test: We tested the null hypothesis of unimodality using Hartigan’s dip statistic, which measures the maximum deviation from a unimodal distribution. The significance threshold was set at p < 0.001.

Kernel density estimation: Nonparametric kernel density estimation of age-at-orchiopexy was performed using a Gaussian kernel. The bandwidth was selected using Silverman’s rule of thumb as implemented in Python version 3.9 (Python Software Foundation, Wilmington, Delaware, US). For the bootstrap analysis, 1,000 resamples were generated at the patient level by sampling with replacement from the original cohort. For each resample, the kernel density estimate was recomputed, the number and locations of modes were recorded, and the distribution of mode counts across resamples was summarized.

Gaussian mixture modeling: Gaussian mixture models were fitted with k = 1, 2, and 3 components using maximum likelihood estimation. Models were initialized with multiple random starts using k-means-based initialization, and the solution with the highest log-likelihood was retained. Convergence was declared when the relative change in log-likelihood fell below 1 × 10⁻⁶ or after a maximum of 1,000 iterations. Analyses were performed using the scikit-learn mixture module in Python version 3.9. Although the two-component model was statistically preferred over one- and three-component models based on BIC, its clinical interpretation as reflecting early and late presentations is hypothesis-driven and should be viewed as consistent with, rather than proof of, the existing two-subtype paradigm.

Secondary analysis

Component Characterization

For the optimal mixture model, we estimated component means, standard deviations, and mixing weights. For the optimal two-component Gaussian mixture model, the intersection age was defined as the solution to the equation $f_1(x) = f_2(x)$, where $f1$ and $f2$ denote the two component density functions. This equation was solved numerically within the observed age range using a root-finding algorithm (e.g., the brentq function in SciPy). Analyses were performed using Python 3.9 (scikit-learn, SciPy) and R 4.1 (diptest package). All code and data are available for reproducibility verification.

## Results

Study population

**Figure 1:**
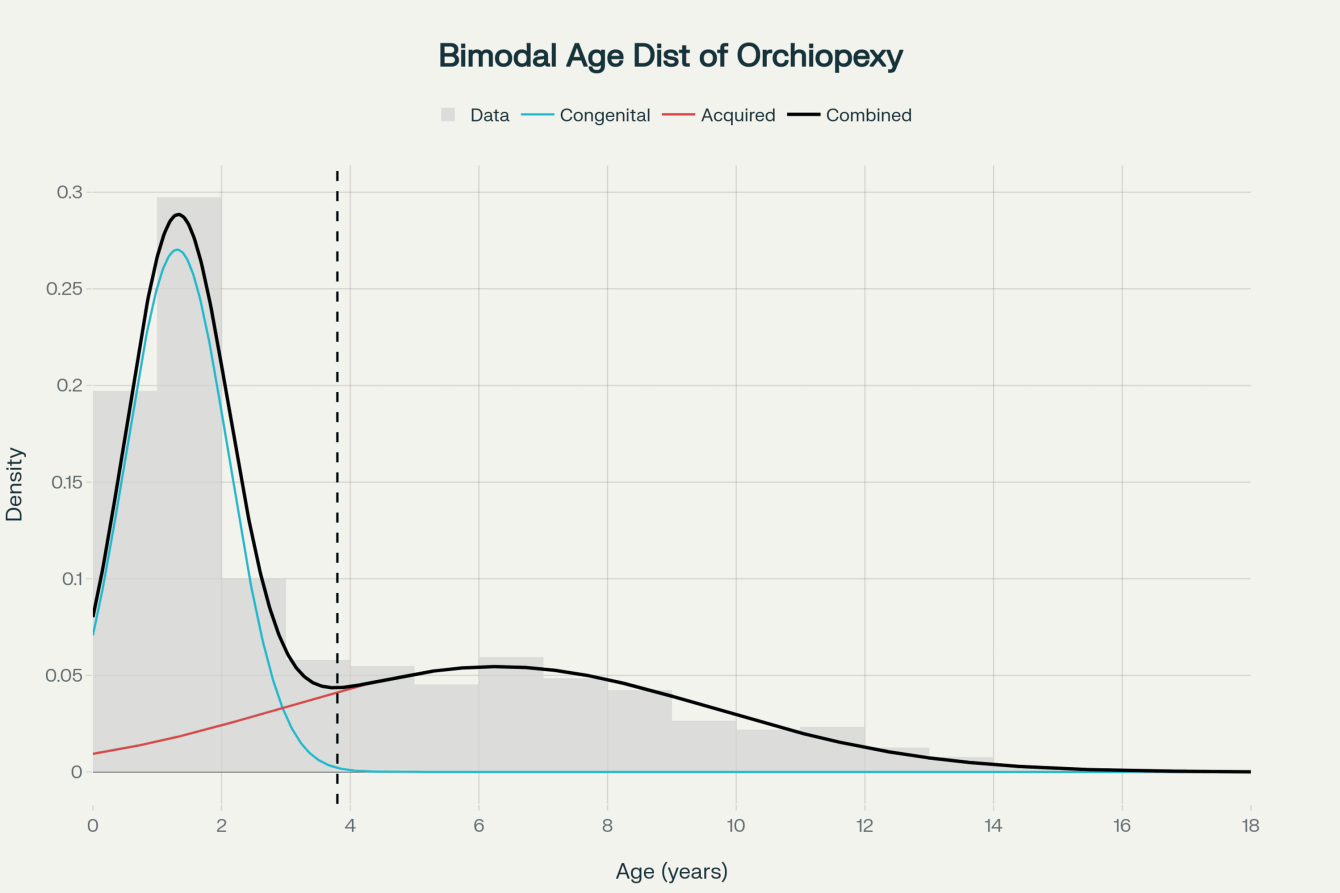
Bimodal age distribution of orchiopexy The histogram displays the age at orchiopexy for 659 patients. The two-component Gaussian mixture model identifies an early component (mean = 1.31 years, weight = 54.2%) and a late component (mean = 6.28 years, weight = 45.8%), with an intersection at 3.8 years. This bimodal pattern is consistent with clinically hypothesized congenital and acquired subtypes, though biological validation remains to be determined.

The dataset comprised 659 orchiopexy cases with ages ranging from zero to 18 years (mean = 3.59 years, median = 2.0 years). Visual inspection of the age distribution suggested potential bimodality with peaks in early childhood and school-going age (Figure 1).

Bimodality testing

**Figure 2:**
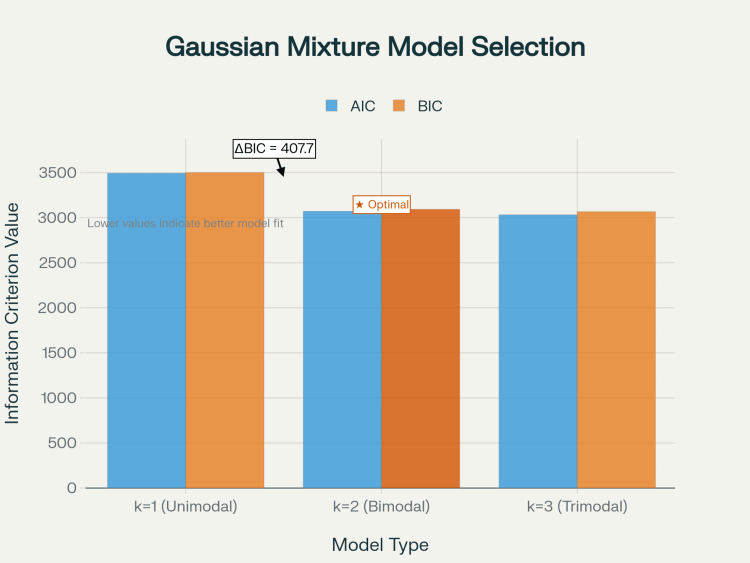
Gaussian mixture model selection for bimodal structure Comparison of information criteria (AIC and BIC) for unimodal (k = 1), bimodal (k = 2), and three-component (k = 3) models. The two-component model (k = 2) shows the lowest BIC value (3093.2) with ΔBIC = 407.7 compared to the unimodal model, providing overwhelming statistical evidence for bimodality in the orchiopexy age distribution. AIC: Akaike Information Criterion, BIC: Bayesian Information Criterion

Hartigan’s dip test decisively rejected unimodality (dip statistic = 0.134, p < 0.001), providing strong evidence against a single-mode distribution. Gaussian mixture model selection was performed with models containing k = 1, 2, and 3 components using maximum likelihood estimation. Model comparison using the Akaike Information Criterion (AIC) [5] and Bayesian Information Criterion (BIC) [6] revealed overwhelming support for the two-component structure (Figure 2).

For the unimodal model (k = 1), AIC was 3491.9, and BIC was 3500.9, while the two-component model (k = 2) demonstrated AIC of 3070.7 and BIC of 3093.2, and the three-component model (k = 3) showed AIC of 3031.4 and BIC of 3067.3. The two-component model showed ΔBIC = 407.7 relative to the unimodal model, indicating very strong evidence for bimodality. While the three-component model yielded slightly lower AIC, BIC strongly favored k = 2 over both the unimodal and three-component models.

Component characterization

The optimal two-component model identified distinct early and late components in the age-at-orchiopexy distribution (Figure 1). The early component had a mean age of 1.31 years (SD = 0.80) and a weight of 54.2%, while the late component had a mean age of 6.28 years (SD = 3.35) and a weight of 45.8%. The intersection between these two components was calculated at 3.8 years. Using this intersection as a cutoff, the early component encompasses 62.7% of cases (ages ≤ 3 years), while the late component includes 37.3% of cases (ages > 3 years). Kernel density estimation with bootstrap resampling (n = 1000) consistently identified two modes near the component means, confirming structural stability of the bimodal pattern.

## Discussion

This study provides strong statistical evidence that the age distribution of orchiopexy procedures is bimodal, in a pattern that is consistent with the clinical paradigm of congenital and acquired cryptorchidism subtypes. However, our findings should be interpreted as supporting this clinical hypothesis rather than as definitive biological proof of distinct disease entities [1]. Three independent analytical approaches, dip testing [7], mixture modeling [6], and bootstrap density estimation, converged to demonstrate a significant departure from unimodal distribution. Bimodal age distributions have been demonstrated in other pediatric and adult disease processes [8], supporting the robustness of our analytical approach in characterizing age distribution patterns.

Clinical interpretation of components

The early component (mean equals 1.31 years) is consistent with congenital undescended testes managed during guideline-recommended surgical timing windows [9] and may predominantly reflect congenital presentations, although this cannot be confirmed without standardized documentation of testicular position at birth and early infancy [10]. The late component (mean equals 6.28 years) is compatible with the clinical concept of acquired cryptorchidism, in which previously descended testes are thought to undergo secondary ascent [11]. However, in this retrospective dataset, we did not have systematic documentation of previous descent, and thus this interpretation should be regarded as hypothesis-generating.

Implications for clinical practice

The intersection at 3.8 years provides a quantitative summary of where the two statistical components overlap and may serve as an exploratory threshold for age-stratified considerations [12]. Cases presenting at younger ages are more likely to align with the early component, whereas those presenting at older ages are more likely to align with the late component. However, this threshold has not been validated against clinical outcomes or prospectively defined subtypes and therefore should not be used as a prescriptive cutoff for individual patient management.

Statistical considerations

The overwhelming statistical evidence (delta BIC equals 407.7) far exceeds conventional thresholds for model preference (delta BIC greater than 10) [13]. This magnitude demonstrates strong statistical support for bimodality in the surgical timing distribution, though its biological interpretation requires prospective validation.

Limitations

Study limitations include several important factors. First, this is a retrospective, single-center study from a tertiary referral institution, and the referral population may differ systematically from community-based presentations, introducing selection bias and limiting generalizability. Second, age at surgery is not equivalent to age at disease onset. Surgical timing is influenced by practice guidelines, referral pathways, socioeconomic factors, parental decision-making, and healthcare access, in addition to underlying disease biology; thus, the observed age-at-orchiopexy distribution reflects both biological and health system factors. Third, we did not adjust for potential confounders such as prematurity, genetic disorders, and birth weight, which may affect both the presentation of cryptorchidism and the timing of orchiopexy. Fourth, we lacked standardized clinical and histopathologic validation to confirm that the two statistical components correspond directly to congenital versus acquired subtypes (e.g., documentation of testicular position at birth, serial examinations confirming ascent, or outcome-based differentiation). Therefore, our interpretation of the early and late components as reflecting congenital and acquired presentations should be considered hypothesis-generating rather than definitive. Future prospective multicenter studies with detailed phenotyping and longitudinal follow-up are needed to formally validate these potential subtypes and to link them to outcomes.

## Conclusions

Age-at-orchiopexy demonstrates statistically robust bimodality with early (1.31 years) and late (6.28 years) components, consistent with and potentially correlating with clinically hypothesized congenital and acquired cryptorchidism subtypes. The intersection at 3.8 years provides a quantitative reference point that may be useful for hypothesis generation and future research design. These findings do not validate the biological basis of the current two-subtype paradigms but provide rigorous statistical support for the observed age distribution pattern and should serve as a starting point for prospective clinical validation.
